# Glyphosate toxicity and carcinogenicity

**DOI:** 10.17179/excli2018-1581

**Published:** 2018-08-16

**Authors:** Tomoyuki Kawada

**Affiliations:** 1 Department of Hygiene and Public Health, Nippon Medical School

## ⁯⁯

Dear Editor,

Tarazona et al. (2017[5]) reviewed the association between glyphosate toxicity and carcinogenicity. They described that the levels of glyphosate exposure were under the reference values based on human bio-monitoring and food residues monitoring. In addition, the low incidence of specific cancer would lead to the lack of statistical power for the analysis. Andreotti et al. (2018[1]) reported that there was no significant association between glyphosate use and subsequent cancer incidence. The present letter discusses these associations.

First, protective equipment against glyphosate exposure would reduce the total amount of load, and the biological monitoring of glyphosate exposure is recommended for the risk assessment (Connolly et al., 2017[3]). Although the biological monitoring of glyphosate exposure would be difficult for large populations, the dose-response relationship between glyphosate exposure and cancer incidence should be evaluated quantitatively in human studies.

Second, Tarone (2018[6]) assessed glyphosate as a probable human carcinogen and mentioned that cancer prevention activities should be based on scientific assessment of carcinogenic agents. In combination with laboratory data on chemical toxicity, Chang and Delzell (2016[2]) conducted a meta-analysis of glyphosate exposure and the risk of leukemia, presenting a pooled relative risk (95 % confidence interval) of 1.0 (0.6-1.5). They included only three studies, and further epidemiological reports are needed for stable estimates.

Finally, Connolly et al. (2018[4]) presented a report on glyphosate exposure levels in adults without occupational exposure. Glyphosate was detectable in 20 % of the samples collected from Irish adults and the widespread use of glyphosate was suspected. The effect of chronic low-dose exposure to glyphosate on human health should also be evaluated.

## Conflict of interest

The author declares no conflict of interest.
